# An inverse free electron laser acceleration-driven Compton scattering X-ray source

**DOI:** 10.1038/s41598-018-36423-y

**Published:** 2019-01-24

**Authors:** I. Gadjev, N. Sudar, M. Babzien, J. Duris, P. Hoang, M. Fedurin, K. Kusche, R. Malone, P. Musumeci, M. Palmer, I. Pogorelsky, M. Polyanskiy, Y. Sakai, C. Swinson, O. Williams, J. B. Rosenzweig

**Affiliations:** 1 UCLA Department of Physics and Astronomy, 405 Hilgard Ave., Los Angeles, CA 90095 USA; 2 Brookhaven National Laboratory, Upton, NY 11973 USA

## Abstract

The generation of X-rays and γ-rays based on synchrotron radiation from free electrons, emitted in magnet arrays such as undulators, forms the basis of much of modern X-ray science. This approach has the drawback of requiring very high energy, up to the multi-GeV-scale, electron beams, to obtain the required photon energy. Due to the limit in accelerating gradients in conventional particle accelerators, reaching high energy typically demands use of instruments exceeding 100’s of meters in length. Compact, less costly, monochromatic X-ray sources based on very high field acceleration and very short period undulators, however, may enable diverse, paradigm-changing X-ray applications ranging from novel X-ray therapy techniques to active interrogation of sensitive materials, by making them accessible in energy reach, cost and size. Such compactness and enhanced energy reach may be obtained by an all-optical approach, which employs a laser-driven high gradient accelerator based on inverse free electron laser (IFEL), followed by a collision point for inverse Compton scattering (ICS), a scheme where a laser is used to provide undulator fields. We present an experimental proof-of-principle of this approach, where a TW-class CO_2_ laser pulse is split in two, with half used to accelerate a high quality electron beam up to 84 MeV through the IFEL interaction, and the other half acts as an electromagnetic undulator to generate up to 13 keV X-rays via ICS. These results demonstrate the feasibility of this scheme, which can be joined with other techniques such as laser recirculation to yield very compact photon sources, with both high peak and average brilliance, and with energies extending from the keV to MeV scale. Further, use of the IFEL acceleration with the ICS interaction produces a train of high intensity X-ray pulses, thus enabling a unique tool synchronized with a laser pulse for ultra-fast strobe, pump-probe experimental scenarios.

## Introduction

The rapid progress in X-ray science over the last century, beginning with cathode ray tubes and arriving at 4^th^ generation light sources – in the form of the X-ray free-electron laser, or XFEL – has been driven by many breakthroughs in the fields of electron acceleration and synchrotron radiation generation. Dramatic advances in these techniques have fueled many discoveries across a wide swath of scientific disciplines ranging from physics^1,2^, to chemistry^3^, biology^4^, and material science^5^. The main drawback of modern high brightness X-ray sources is their large size and cost, driven both by the size and complexity of the high energy particle accelerators and the elaborate undulator magnets used. In an attempt to create a more compact, high flux, high brilliance X-ray source, 5^*th*^ *generation X-ray sources* based on all-optical schemes involving laser-driven accelerators and laser-enabled undulators have been the subject of intensive study^6–11^.

In such an all-optical X-ray light source, one may replace the undulator or wiggler magnets, which have cm-scale period, with the *μ*m-wavelength oscillating electromagnetic field of a high power laser. This change, to a relativistic electron-intense laser radiative interaction, is termed inverse Compton scattering (ICS), allows one to reach similar photon energies as reached by magnetostatic undulators with two orders of magnitude lower energy electron beams. For example, at the Brookhaven National Laboratory’s Accelerator Test Facility (BNL ATF), site of the experiments reported here, tens of MeV-scale electrons can scatter far-infrared CO_2_ laser photons to reach X-ray energies in the tens of keV^12,13^. Further, the ICS approach is uniquely suitable for the production of very high-energy photons. With GeV-class electron beams, one may produce high brilliance, spectrally narrow γ-ray beams, with photon energies in the MeV range and beyond^14^. Given such possibilities, an ICS-based X-ray source possesses desirable characteristics for many X-ray applications that demand narrow bandwidth, short pulse, and directional high-flux X-ray beams.

While taking advantage of the ICS interaction greatly reduces the demands on the electron beam, in order to reach MeV-class photon energies and also improve the brilliance of the X-rays, an electron beam with several 100’s of MeV energy is still needed. To this end, it is possible to use the same laser pulse utilized for the Compton interaction to enable a compact, high-gradient electron accelerator based on the inverse free-electron laser (IFEL) scheme. An IFEL has the demonstrated capability to produce notably higher than state-of-the-art acceleration gradients in a material free interaction region^15^. It thus permits use of the laser for acceleration without the conventional limitations arising from nearby matter in accelerators, *i.e*. wakefields, and material breakdown at high field. Indeed, the IFEL has been shown to be a highly efficient process that does not significantly deplete the driving laser pulse^15,16^ for electron beam currents below 10 kA. This quality sets IFEL acceleration apart from high gradient laser plasma accelerator (LPA) schemes, as in the IFEL the high-power laser pulse used can be recirculated through an amplification scheme and thus achieve burst-mode repetition rates in the >10 MHz range. Laser recirculation, paired with an electron beam pulse train, has been demonstrated for an ICS source by Ovodenko *et al*.^17^ and the effort is now being extended to demonstrating this technique in an IFEL.

There is strong global interest in the development of 5^th^ generation light sources – the use of advanced accelerators to obtain high brilliance keV-to-MeV photon beams, with many projects now being initiated^18^. In context of the present work, given the rapid progress in recent years on the physics of both ICS and IFEL, and the recent demonstration at the BNL ATF of ICS enhanced by laser recirculation, it is quite timely to examine the intersection of ICS and IFEL in a single experiment. In this paper, we show the use of an IFEL that produces a high quality, micro-bunched accelerated electron beam which in turn feeds a Compton scattering interaction point (IP) based on the same laser system, for the production of a narrow bandwidth, directional, pulsed X-rays. We thus demonstrate a unique all-optically driven, electron beam-based X-ray source. This is an important step forward in the burgeoning research into advanced accelerators and their use in real world applications, in particular compact light sources. The experimental challenges encountered required addressing frontier demands in accelerator physics concerning beam quality^19^ and have pushed forward experimental techniques needed to arrive at yet higher energy applications of advanced accelerators^20^.

## Background

We first review some basic properties of the ICS process. Photons generated by ICS are localized in angle to a *θ* ~ 1/*γ* cone about the electron propagation direction – a directionality characteristic of radiation by relativistic charged particles. For ultra-relativistic electrons, the Lorentz factor $\gamma =\frac{{U}_{e}}{{m}_{e}{c}^{2}}\gg 1$, is the ratio of the electron energy, *U*_*e*_, to its rest mass, *m*_*e*_*c*^2^. Neglecting the recoil of the electron (the Thomson limit, which is valid when the laser photon’s momentum in the electron rest frame is smaller than the electron’s rest energy), the single-particle angular dependence of the X-ray spectrum is: $\frac{h{k}_{x{\rm{ray}}}}{h{k}_{L}}=\frac{4{\gamma }^{2}}{1+{\gamma }^{2}{\theta }^{2}}$. While there is an inherent off-axis redshift, the distribution in *θ* is confined to within this 1/*γ* angle; these higher energy components thus also correspond to the highest flux density of X-rays.

In practice, the scattering takes place in the context of a highly focused, short pulse beam of electrons colliding with a laser pulse of similar spatio-temporal characteristics. There are a number of aspects of the interaction arising from the distribution of electron and photon angles in the beams, as well as the influence of the finite time of laser-electron interaction, that affect the flux, bandwidth, and divergence of the X-ray photon distribution generated^21^. The most basic of these considerations is that the total number of generated X-rays is proportional to the number of electrons and laser photons available for interaction as well as the cross-sectional overlap of the two beams, *i.e* the luminosity *L* of the collision. If the transverse profiles of the electron bunch and laser pulse are well approximated by cylindrically symmetric bi-gaussian distributions, the optimally efficient configuration is obtained when the electrons and laser have the same transverse size, *σ*_*L*_ = *σ*_*x*_, giving ${N}_{xray}={\sigma }_{T}{N}_{e}{N}_{L}/4\pi {\sigma }_{x}^{2}={\sigma }_{T}L$, where *σ*_*T*_ is the Thomson cross-section, *N*_*e*_ and *N*_*L*_ are the number of electrons and laser photons present in the scattering event.

The ICS process is also equivalent to using an optical undulator; here we combine it with an optical accelerator, the IFEL, which is an efficient process for transferring energy from a laser pulse to a co-propagating relativistic electron beam^22^. In present radio-frequency accelerators, boundary conditions are employed that permit rotation of the electric field to have a significant longitudinal component, and thus energy can be exchanged with particles traveling in the longitudinal direction. If one wishes to use lasers, ubiquitous sources of very high electromagnetic power, to accelerate electrons, however, the acceleration process must be re-examined. Given the small dimensional scale and high fields of the wave used, vacuum acceleration techniques are advantageous. The IFEL is such a scheme, in which for energy transfer to occur the transverse electric field of the laser pulse is coupled to the electron motion via the transverse oscillation induced by a periodic magnetic field. In order to have continuous acceleration in an IFEL, a stationary phase of the electrons with respect to laser pulse must be maintained^23^. This is managed in practice by adjusting the period of the external magnetic field, a procedure termed undulator “tapering”. Inside an undulator, the electrons with resonant energy, maintain a nearly constant interaction phase with co-propagating light waves (wavenumber, *k*_*L*_), when the undulator condition is met: $\frac{{k}_{u}}{{k}_{L}}=\frac{1+{K}_{u}^{2}}{2{\gamma }^{2}}$. As the electron energy changes during acceleration to maintain resonance one should taper the undulator so as to continuously preserve the accelerating phase of the ponderomotive force. The resonance condition indicates that one may change either the normalized undulator vector potential, *K*_*u*_ or the undulator period, *λ*_*u*_ = 2*π*/*k*_*u*_, or both, to accomplish this goal.

**Figure 1:**
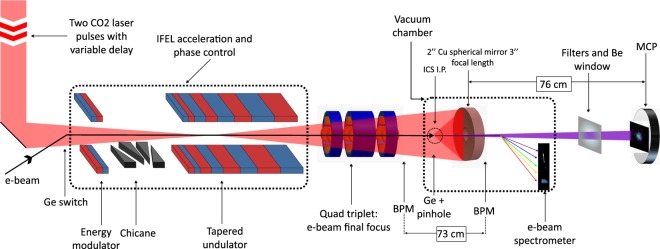
Schematic of the experimental layout, showing electron and laser beamlines. The two CO_2_ laser pulses co-propagate with the electron beam and are reflected by the spherical Cu mirror. The electron beam is imaged onto the spectrometer after colliding with the leading CO_2_ pulse. The generated X-rays are collected by an MCP, permitting a spatially resolved measurement.

Although IFEL acceleration gradients are modest compared to an alternative laser-based acceleration concept, laser plasma acceleration (LPA)^24^, the IFEL approach has a number of important advantages. First, the IFEL is as noted a free space accelerator (*cf*. Fig. 1), and as such no energy is lost to the media, and very high gradients can be sustained without a risk of material breakdown – as in, e.g. a dielectric laser accelerator^25^. With the ability to use weakly focused, very high energy laser pulses, a single IFEL stage can be made up to a few meters long, providing continuous interaction over extended lengths. Further, absorption of energy from the electron beam is the only notable effect of the interaction that influences the radiation propagation. Therefore, for low electron beam currents, pump depletion effects can be compensated, through recycling the laser beam in a high repetition rate configuration^26,27^. This configuration enables greatly enhanced average radiation flux, which is an over-arching goal of the research described here, demanded by applications ranging from medicine to non-intrusive inspection. Finally, since the resonant characteristics of the IFEL interaction are (assuming a laser with modest intensity fluctuations) dominated by the static magnetic fields of the undulator, the accelerated beam longitudinal phase space can be well controlled through proper undulator design. This process can result in small energy spread and much reduced output energy fluctuations^22^, especially compared to schemes such as LPA which translate laser intensity errors to electron output energy errors^19^. We note that the phenomenon of energy loss to wakefields has an analogy in the IFEL – synchrotron radiation losses. These effects limit the maximum practical beam energy in an IFEL to the 10’s of GeV range, which is does not present an obstacle to light source applications.

## Results

This merging of the IFEL accelerator and the ICS X-ray IP to yield a unique source of X-ray photons was performed on a high brightness electron beamline at the BNL ATF. The general features of this beamline, which permits electron interactions with a high peak power CO_2_ laser operating at *λ*_*L*_ = 10.3 µm, are shown in Fig. 1. Electron bunches with total charge of 300 pC and a duration of 3 ps are generated by a Cu cathode photo-injector at a rate of 1 Hz. The electrons are accelerated by a couple of RF accelerating sections to a nominal energy of 52 MeV. These and other important electron beam and laser parameters are catalogued in Table 1. There are three points of laser-electron interaction in this experimental scenario: a short IFEL energy modulator is first encountered, which combined with a downstream chicane forms a pre-bunching system; a subsequent tapered IFEL undulator, known as the Rubicon undulator, is where the laser provides ponderomotive acceleration; and, finally, the ICS interaction point yields X-ray production. This experimental design requires two co-linear laser pulses obtained from the same CO_2_ laser amplifier. The pre-bunching and IFEL interaction sections are driven with the trailing CO_2_ pulse. The leading CO_2_ pulse, on the other hand, is reflected and re-focused onto the electron bunch by a spherical Cu mirror, *f*/# = 1.5, which is housed in a vacuum chamber 1.8 m downstream of the undulator. The ~0.5 ns delay between the two laser pulses is nominally double the time of flight associated with the Cu mirror focal length (*f* = 7.5 cm). An electron beam activated Ge switch was inserted directly upstream of the pre-buncher, and the CO_2_ transmission through the Ge screen was used to superimpose the electron beam and laser in time to ~1 ps resolution^28,29^. Finer, sub-picosecond level synchronization was achieved based on optimization of the acceleration and the X-ray flux. Immediately after the ICS IP, the electron beam is deflected by a permanent magnet dipole and dumped onto a large energy acceptance spectrometer. The Compton X-rays are detected 76 cm downstream using a micro-channel plate (MCP), which gives not only the total flux, but the angular distribution of these ICS-derived photons.

**Table 1:** The nominal and averaged measured parameters associated with the electron beam (before and after IFEL acceleration), the CO_2_ laser, and the ICS X-rays are presented here for convenience.

Parameter	Unit	Value	
*Electron beam*		*Initial*	*After IFEL*
Energy, *U*_*e*_	MeV	52.0	81.7
Energy spread, $\delta \gamma =\frac{{\sigma }_{\gamma }}{{\gamma }_{0}}$	—	<0.005	0.028
Charge, *Q*_*e*_	pC	250	100
Bunch length, *σ*_*t*_	ps	3	2.5
Normalized emittance, *ε*_*n*_	mm-mrad	2.3 ± 0.1	2.4 ± 0.15
Transverse size at ICS IP, *σ*_*x*_ × *σ*_*y*_	µm	140 × 170 (±10)	140 × 170 (±10)
Repetition rate	Hz	1	0.008
***CO***_**2**_ ***Laser Pulse***			
Central wavelength, *λ*_*L*_	µm	10.3	
Energy, *U*_*L*_	J	1.02 ± 0.12	
Pulse length, *σ*_*t*_	ps	2.5	
Transverse size at ICS IP, *w*_0_	µm	150	
Repetition rate	min	1–2	
***ICS X-rays***			
Central energy	keV	11.6	
Photons per shot	#	1.21 (±0.63) × 10^6^	
Opening angle, *θ* ~ 1/*γ*	mrad	3.4 ± 0.1	

The main features of the Rubicon undulator have been documented in previous publications by Duris *et al*.^22^. The undulator is a strongly tapered, helical, permanent magnet design that permits efficient, continuous accelerating and bunching interaction between the electrons and the co-propagating laser. It is the first of its type deployed for such experiments.

**Figure 2:**
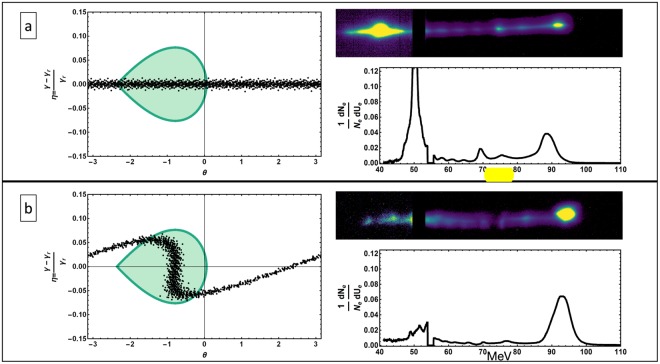
The left side figures present the electron beam longitudinal phase-space distribution overlaying the stable phase-space area due the accelerating potential. The shaded area in the (*η*, *θ*) plots signifies this stable phase-space that is captured and subsequently accelerated. The electron spectrometer images and lineouts, in the right side figures, show the significant increase in capture efficiency when the pre-buncher is employed to load the initial electron beam distribution centered at − *π*/4 phase of the ponderomotive potential.

To increase the fraction of accelerated electrons captured in the Rubicon IFEL’s longitudinal acceptance, we employed a pre-buncher assembly to shape the longitudinal phase space of the electron beam upon entrance into IFEL^30,31^. The pre-buncher consists of a single period permanent magnet planer undulator and a permanent magnet variable-gap chicane and is described by Sudar, *et al*.^32^. The laser’s electric field imparts periodic energy modulation on the beam as the two co-propagate inside the single period undulator. This energy modulation is then transformed to a density modulation, or microbunching through use of a chicane magnet system, that also controls the electrons’ position relative to the phase of the laser electric field. Placing the peak of the density distribution at the appropriate ponderomotive IFEL phase significantly enhances the capture fraction. The longitudinal phase-space plots in Fig. 2 portray representative electron distributions and the region in (*η*, *θ*) space that is captured to full acceleration by the ponderomotive IFEL potential. The results shown in Fig. 2 demonstrate a clear increase in the fraction of electrons accelerated. The pre-bunched IFEL (b) accelerated 42% of the beam as compared to only 17% in the non-pre-bunched IFEL case (a). It should be emphasized that both the relatively modest bunching and energy gain used in these experiments – both well under the maximum performance limit demonstrated using the Rubicon system – are a consequence of the experimental requirements that needed to be met to use the same laser system not only for the IFEL, but also the ICS interaction. They are specific to this scenario, and not indicative of the ultimate performance of an IFEL-driven ICS source.

**Figure 3:**
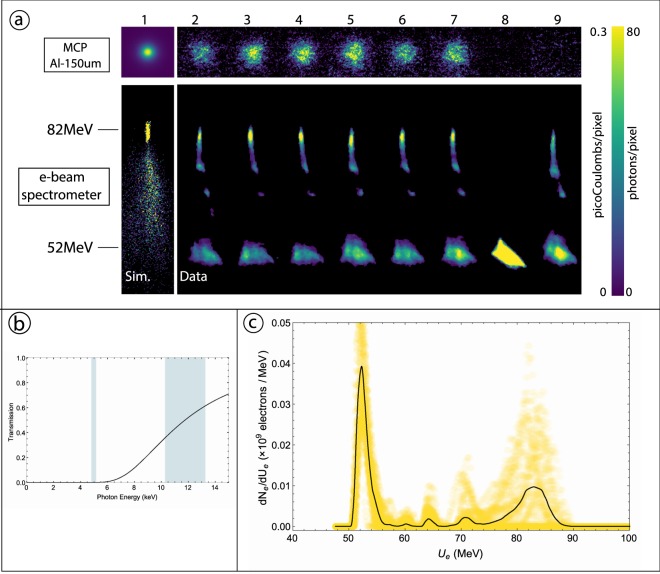
(**a**) Images obtained at the electron spectrometer and the X-ray MCP. Higher energy electrons appear towards the top of the spectrometer images. The MCP images of the detected X-rays are taken downstream of a 150 *μm* thick Al attenuator, whose transmission curve is shown in part (b). The shaded bands in (**b**) indicate the ranges of central energies of the X-rays produced by electrons from the 52 MeV and 82 MeV peaks of the electron spectrum in part (c). (**c**) Overlays of 44 electron beam spectra taken during IFEL operation, with the average spectrum (solid black line). The sequence of six representative shots in which both IFEL acceleration and ICS X-rays were observed is given in images 2–7. Image 8 shows an unaccelerated electron beam, which salso yields ICS X-ray flux completely attenuated by the Al filter. Image 9 shows the X-ray background from an accelerated e-beam. The 3D simulation of the IFEL process and the expected X-ray signal in image 1 is in good agreement with the data presented.

The results of merging the IFEL bunching and accelerating systems with the IFEL interaction system show aspects of the new class of light source enabled by this synthesis of techniques. To this end, electron spectra and X-ray angular distributions from a sequence of six measurements are displayed in part a) of Fig. 3. Images from the electron energy spectrometer are shown in the bottom figures, with higher energy electrons appearing towards the top of each image. The electron beam was bunched and accelerated by the IFEL from 52 MeV to a maximum of 84 MeV. Part c) of the figure plots the measured energy distributions of 44 electron beams after IFEL acceleration, with the average distribution graphed in a solid black line. The mean of the energy peak of the accelerated electrons is 81.7 MeV with a pulse-to-pulse rms deviation of 0.5 MeV, which is notably smaller than the rms energy spread of the peaks, which is 2.3 MeV, or 2.8%. The total energy gain was over 30 MeV, corresponding to an effective maximum accelerating gradient in the IFEL of 55 MeV/m for correctly phased electrons. The final spectral center energy is stable against laser fluctuations, as illustrated by the collection of shots shown in Fig. 3 and by the rms value quoted above In fact, the final electron beam energy is dictated, within certain bounds of intensity, by the undulator design. However, the fraction captured at the final energy is susceptible to fluctuations in alignment, energy, and timing of the laser pulse. This fluctuation can be seen in the variation of the height of the 82 MeV peak in Fig. 3c). While obtaining high capture rates is not the main emphasis of this experiment, it should be noted that a follow-up experiment by Sudar *et al*.^33^ was able to accelerate 90% of the electron beam. It should also be noted that the electron beam longitudinal phase space after IFEL acceleration is dominated by the effects of the IFEL interaction; in addition to energy gain, the beam is micro-bunched at the laser period, and the energy spectrum displays a corresponding increased spread.

**Figure 4:**
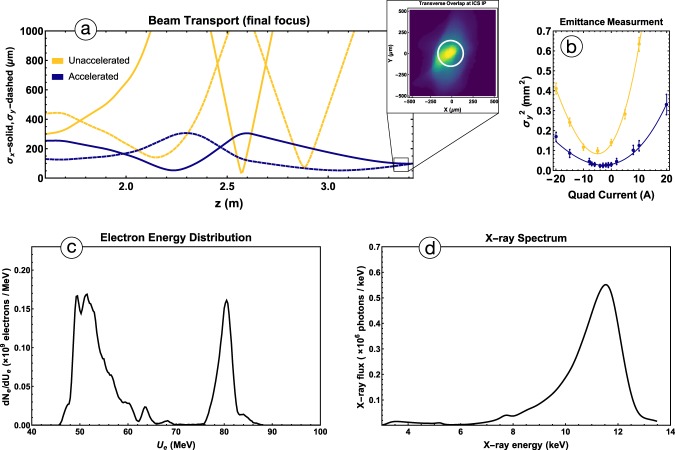
(**a**) The transverse beam envelopes *σ*_*x*_ and *σ*_*y*_ for both accelerated and unaccelerated beams The emittance measurement shown in (**b**) is used as input for particle tracking, in the (**a**), (**c**) IFEL simulated electron energy spectrum. Although the spectrum shows a large population of unaccelerated electrons, the focus into the ICS IP (see (**a**)) is set to produce a transverse focus only for higher energy electrons. This translates into a simulated X-ray spectrum, shown in (**d**), that is dominated by 12 *keV* photons. The inset of (**a**) is the transverse profile of the electron beam at the ICS IP *σ*_*x*_ × *σ*_*y*_ = 140 × 170(±10) *μm*; the superimposed circle marks the estimated CO_2_ FWHM at the IP ~ 150 *μm*. The measurement in (**b**) confirms the preservation of the beam normalized emittance during the IFEL acceleration. Emittance measurement data was taken for both the unaccelerated beam and accelerated beam, yielding normalized emittance values of 2.3 ± 0.1 *μm* and 2.4 ± 0.15 *μm*, respectively. The error bars are calculated based on the resolution of the transverse position monitors and the statistics of the measurement.

In displaying the spectrometer images in part a), we note that the quadrupole triplet’s focusing effects are adjusted to focus the more energetic and rigid accelerated electron beam, and are thus too strong for the non-accelerated beam. The 82 Mev electrons are optimally focused while 52 MeV electrons have passed through a focus and appear diffuse at the spectrometer; they are also not widely distributed at the IP, and thus do not measurably participate in the ICS interaction. This is apparent in the simulation based X-ray spectrum of part d) of Fig. 4, which lacks a peak at 5 keV. The narrow energy band of captured and accelerated electrons is found to suffer negligible emittance growth, a feature of optimized IFEL systems^22^ that is essential for the effective focusing of electrons at the ICS IP. The preservation of e-beam emittance and its effect on the final focus into the ICS IP are reflected in the measurement shown in Fig. 4(b) with a detailed discussion given in the Methods.

The top images in part a) of Fig. 3 show the spatial distribution of ICS X-rays deposited on the MCP after passing through a 150 μm thick Al foil. The Al foil acts as a highly effective attenuator for lower energy X-rays, as indicated by the transmission curve plotted in part b) of Fig. 3. Since the energy distribution of the Compton scattered X-rays reflects that of the electron beam, the portion of X-rays that passes through the Al foil corresponds to X-rays produced from the accelerated, 82 MeV electrons. In part a) of Fig. 3, shots 2–7 indicate clear ICS X-ray production above 10 keV from the accelerated electrons. When the IFEL acceleration is turned off, the Al attenuator blocks all resultant ICS X-rays (image 8). On the other hand, if the ICS laser pulse is not present while IFEL acceleration is present, there is no significant X-ray background to the MCP (image 9). This spectral filtering thus demonstrates successful operation of the IFEL driven ICS X-ray source. The measured X-ray flux extends over the range 10–13 keV, with a central peak at 11.6 keV, and contains 1.21(±0.63) × 10^6^ photons per shot, which is in agreement with the simulation^34^ based estimate of 1.3 × 10^6^ photons per shot.

The temporal characteristics of this source are of a unique form. In head-on ICS collisions, the temporal structure of the X-ray pulse closely follows that of the relativistic electron beam. Further, it is known that both the FEL and IFEL processes produces a micro-bunched electron beam at the resonant wavelength^35,36^. Thus the IFEL used to accelerate an electron beam in preparation for an ICS interaction produces bursts of X-rays spaced at the resonant laser wavelength, *λ*_*L*_ = 10.3 μm  = 34.4 fs. The rms width, which we estimate to be on the order of 2 fs, is defined by the size of the ponderomotive potential. While there was no direct measurement of the electron beam microbunching in this experiment, it will be possible in the future to do so through use of a fs-resolution RF deflector scheme. These bursts of X-rays are incoherent, with a peak brightness estimated to be 2.1 × 10^22^ photons s^−1^ mm^−2^ mrad^−2^ at 0.1%BW. This value compares to the leading storage-ring X-ray sources like the NSLS-II and is well suited for applications such as phase-contrast imaging^37^, but is orders of magnitude below XFELs such as the LCLS. The IFEL-ICS’s differentiating feature with respect to storage-rings is the pulsed temporal structure of the X-rays, which is produced, albeit at much lower brightness, in coherent HHG sources. Finally, the possibility of extending the radiation photon energy of an IFEL-ICS source into the multi-MeV range, a spectral region inaccessible to FELs, makes this a unique light source.

**Figure 5:**
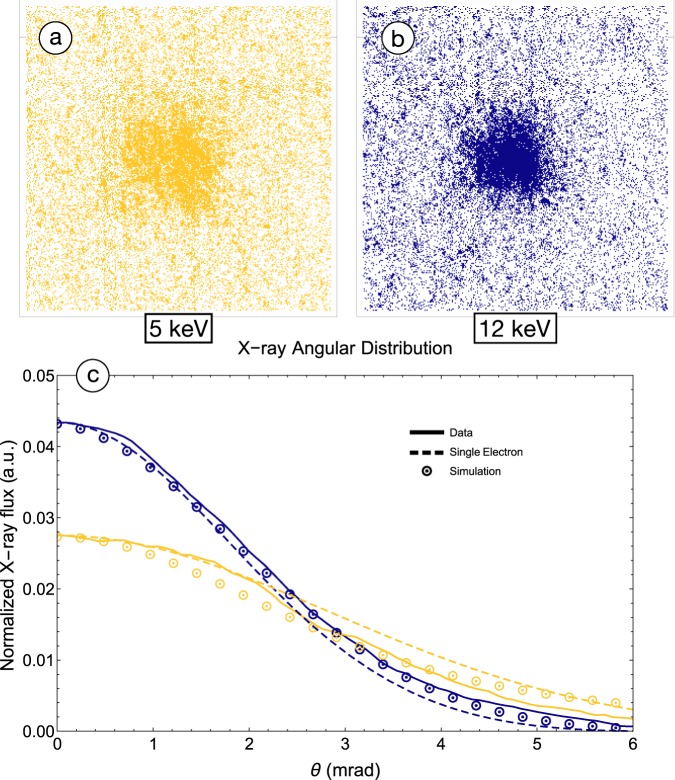
The images in (**a**) and (**b**) are false-color raw images collected from the measuremnt MCP intensity distributions. The angular distribution of X-rays has a characteristic opening angle that depends on the energy of the electrons involved in the Compton scattering process, *θ* ~ 1/*γ*. Harder X-rays are produced by higher energy electrons and have a smaller opening angle, 3.4 ± 0.1 mrad. This is demonstrated in (**c**), where the lineouts from (**a**) and (**b**) are compared to a Compton scattering simulation and the analytic expression for a single electron emission. The lineout amplitudes are normalized to give equal areas.

As a further validation of both the electron beam energy increase and the concomitant increase in X-ray brightness obtained with the use of IFEL acceleration, we examine the angular distribution of the scattered photons. The ICS X-ray distribution exhibits a *θ* ~ 1/*γ* forward opening angle typical of the radiation by an accelerated relativistic particle. This property can be used to deduce the energy of the emitted X-rays by measuring their angular spread, assuming the angles in the electron beam at the IP are <1/*γ*. Figure 5 shows the distribution of X-rays on the MCP detector for two different energies: 5 keV X-rays produced by the unaccelerated electrons at 52 MeV without an attenuating foil, and 12 keV X-rays produced by the fully accelerated electrons at 82 MeV passing through a 150 *μ*m thick Al attenuating foil. The lineouts through the center of these transverse profiles clearly show the narrowing of the angular spread for higher energy X-rays. The angle at which 90% of the flux is encompassed for the 5 keV X-rays occurs at *θ*_52_ = 4.7 ± 0.1 mrad, while for the 12 keV flux this angle narrows to *θ*_82_ = 3.4 ± 0.1 mrad. This observation validates the increased energy in the electron beam, and also illustrates that this beam retains its small angles after IFEL acceleration and transport to the IP — a limit on beam emittance growth is set.

## Discussion

The results presented here show the successful merging of an IFEL accelerator with an ICS interaction point, to yield a first demonstration of a new class of all-optical X-ray light source. This source, owing to its unique dynamics properties, operated with a stable shot-to-shot electron beam and X-ray characteristics. The maximum acceleration gradient achieved in the IFEL of 55 MeV/m, obtained at laser intensities near 10^13^ W/cm^2^ already exceeds that utilized in nearly all existing RF linear accelerators. Furthermore, the use of an optical pre-buncher enabled the fraction of accelerated electrons to be ~40%, a number which has been in subsequent experiments increased to over 90%, as discussed in Sudar *et al*.^33^. These accelerated electrons were used to create 11.6 keV X-rays with a photon yield of 1.21(±0.63) × 10^6^ per shot. It should be noted that not only is the method of creating these X-ray pulses unique, but they also have novel, heretofore unrealized properties owing to the temporal structure of the accelerated beam that produces the X-rays. The IFEL produces a distinctive micro-bunched electron beam, which has density spikes at the wavelength of the driving laser, in this case CO_2_ at 10.3 μm. The temporal structure of the X-ray spectrum closely mimics that of the electron beam and in this way forms a train (34 fs period) bursts of photons. This periodic burst pattern is also produced in HHG sources^38^ in the soft-X-ray spectral region, and has found compelling use in the stroboscopic probing of periodically driven (e.g. by a pulse of the source laser itself) physical systems^39^. The ICS source extends the spectral range such methods to hard X-rays and beyond, and at much higher peak intensity.

The *proof-of-principle* experiment executed here establishes the IFEL acceleration scheme on solid footing as a driver for a 5th-generation, short wavelength light-source. It has been shown that the IFEL may indeed be applied as a compact, moderate-energy, optical accelerator that may serve as a driver for a flexible, compact, laser-electron interaction-based source that accesses a highly useful range of photon energies, extending with accessible experimental parameters from 0.01 to 10 MeV. As such, the results of this experiment are a promising step forward in advanced accelerator and light-source research, showing a novel approach that may be exploited to produce a short wavelength photon source having a number of desirable characteristics, including compactness, the potential to reach very high fluxes through recirculation, and the capability of producing femtosecond, periodic, stroboscopic X-rays.

## Methods

### Two pulse amplification

The staging of the IFEL accelerator and the Compton scattering IP requires two high-power CO_2_ laser pulses separated by 500 ps, both of which are obtained from the terawatt CO_2_ laser system at the BNL ATF. In order to create two laser pulses, the seeding pulse to the main amplifier was split prior to amplification. This was done with a Michelson interferometer type of beam-splitter, which output two co-propagating pulses, each with 25% of the original energy. These two seed pulses were individually amplified by a single discharge of the main amplifier. The energy extracted by each pulse from the main amplifier scales linearly with the energy of the seeding pulse. The output laser energy per pulse was stable with an average energy of 1.02 ± 0.12 *J*. The IFEL acceleration gradient and the capture rate have a direct dependence on the magnitude of the electric field of the driving laser, as discussed in Duris, *et al*.^22^. The tapering of the undulator is governed by practical requirements on the capture rate and the acceleration gradient for a given amount of laser energy. At 1 *J* laser energy per pulse, the undulator tapering was tuned to obtain 40% nominal capture rate at an acceleration gradient of 55 MeV/m. The data-set of Fig. 2 was taken with a laser pulse energies 4 times larger than those used for the staging experiments. To counteract the decrease in electron capture due to lower laser energy, the tapering of the undulator was changed to shift the IFEL resonant phase. While this shift in resonant phase increases capture rates, it also leads to a reduction in the accelerating gradient of the IFEL. This interplay between the available laser energy and undulator tapering lead to reduction of the IFEL accelerating gradient in the IFEL-ICS staging experiments, producing electrons with final energy of 82 MeV.

### Spatial Overlap

Maintaining spatial overlap between the electron bunch and the CO_2_ laser is imperative for sustaining the IFEL acceleration and for maximizing the ICS X-ray flux. To ensure proper overlap over the length of the undulator, the individual magnets of the undulator were tuned so that the trajectory of on-axis electrons is completely within the FWHM of the laser for the entire IFEL interaction. The tuning process relies on Hall-probe scans of the on-axis undulator field, which are then compared to a 3D magnetostatic simulations. It is important to keep the angle of exiting electrons close to zero, in order to transport the beam downstream. The electrons’ exit trajectory is heavily influenced by both the final undulator magnets and the alignment of the undulator with respect to the propagation axis of the electron beam. An alignment He-Ne laser was used to define the electron beam axis and the undulator was aligned to this laser. To verify the transverse alignment of the undulator, beam position monitors (BPM) that reveal the transverse location of the He-Ne and e-beam were installed at the entrance and exit of the undulator.

### Final focusing for ICS

The number of scattered X-ray photons is proportional to the densities of the colliding electron bunch and CO_2_ laser pulse. Therefore, a tight focus at the collision point would increase the flux of x-rays produced for a single collision event. On the other hand, the operation of the IFEL requires a significant laser electric field. Because the two CO_2_ pulses have the same path and pass through the same optics, the optics must be arranged in such a way that the laser comes to a waist at two points in its path. The first focus is at the IFEL undulator. A NaCl lens with focal-length 4.5 m was used to achieve a 0.91 mm waist at the IFEL undulator. The leading CO_2_ pulse is refocused and reflected back onto the electron bunch path by a Cu spherical mirror with a *f/#* = 1.5 focal number. Depending on the divergence of the CO_2_ at the mirror, we estimate that the focus at the ICS IP has a waist of between 100 and 150 *μ*m.

In order to focus the electron beam down to a comparable size, 1.8 m downstream of the undulator, a quadrupole triplet was employed. The transport from the end of the IFEL undulator through the focusing triplet and into the ICS IP can be written in terms of the initial beam σ-matrix and an effective transport matrix for the section, $\overline{{\sigma }_{2}}={M}_{1\to 2}\overline{{\sigma }_{1}}{M}_{1\to 2}^{\dagger }$. The transport matrix for the quadrupole -triplet depends on the individual quadrupole strength parameters, ${K}_{q}=\frac{e{\partial }_{x}{B}_{y}}{\gamma \beta {m}_{e}c}$, which in turn are proportional to the magnetic field gradient and momentum of the electron. Therefore, if a beam with a certain emittance and energy is brought to a waist some distance away from the triplet, then a beam with the same emittance, but higher in energy will be focused at the same distance, if the quadrupole strengths are scaled by the ratio of the energies. In these experiments, the quadrupole-triplet was used to focus the 52 MeV beam to a transverse size of *σ*_*x*_ × *σ*_*y*_ = 140 × 170(±10) *μ*m and then the quadrupole strengths were scaled by a factor of 82/52 to focus the accelerated electrons. The achromatic effect of the final focus is clearly observed in the spectrometer images in Fig. 3, where the in-focus portion of the electron distribution is of higher energies. The scaled quadrupole currents brought the higher energy electrons to a focus at the IP, while lower energy electrons were strongly overfocused at the IP. This served to optimally increase the scattered flux of X-rays from higher energy electrons and strongly decrease that of lower energy electrons. The ability to tune the final focus of the electron beam can in this way be used to suppress the production of lower energy X-rays.

In parts a), c), and d) of Fig. 4, a start-to-end simulation of the pre-bunching, IFEL acceleration, final focus, and Compton scattering is presented. Part a) shows the electron beam transport and transverse focus into the ICS IP for the unaccelerated (yellow) and accelerated (blue) components of the electron beams. Since the final focusing quadrupole triplet is optimized for the accelerated beam, the low energy electrons do not contribute notably to the ICS X-ray spectrum, even though they make up a significant portion of the electron beam, as shown in Fig. 4(c,d). It is possible to focus the accelerated electrons into the ICS IP, because of the IFEL’s unique ability to produce beams with a well-defined transverse emittance. In part b) of Fig. 4, quadrupole scan measurements made after the IFEL undulator confirm that electron beam’s normalized emittance is well-preserved during the IFEL acceleration. The normalized transverse emittance of the unaccelerated beam was measured to be *ε*_*n*_ = 2.3 ± 0.1 *μm*, while that of the accelerated beam was measured to be *ε*_*n*_ = 2.4 ± 0.15 *μm*. By properly matching the incoming electron beam envelope to the natural focusing of the IFEL undulator, we demonstrated for the first time conservation of the incoming beam emittance in the IFEL acceleration scheme.

### X-ray detection and filtering

The micro-channel plate (MCP) detector used to image the ICS X-rays has a KBr photocathode that converts X-ray photons to free electrons at a 50% quantum efficiency^40–42^. The active area of the MCP has a radius of 20 mm. It is placed 76 cm after the ICS interaction point, giving an angle of acceptance, ${\theta }_{MCP}=26.3\,{\rm{mrad}} > {\theta }_{X-\mathrm{ray}}=\frac{1}{\gamma }=6.25\,{\rm{mrad}}$. In our experimental conditions, the energy distribution of the Compton scattered X-rays follows that of the electron beam. It is thus possible to infer the X-ray spectrum based on the electron spectrometer data coupled with a knowledge of the electron beam focus. Figure 4(d) shows the simulation reconstructed X-ray spectrum corresponding to a typical IFEL electron beam. In practice, to estimate the flux of higher energy ICS X-rays, a thin Al foil was used to attenuate photons with energies below 6 keV.

### Microbunching measurements

The microbunching induced in the electron beam by the IFEL acceleration produces a periodic structure in the longitudinal dimension of the beam with a period equal to the resonant wavelength. The level of microbunching can be measured by using coherent transition radiation (CTR) techniques^36,43^. However, the CTR wavelength will be the same as the IFEL seed laser making separate detection of the CTR signal impractical in our beamline layout. The beam’s longitudinal profile could be imaged using an RF deflector, but the standard resolution on such a device is not high enough to resolve microbunching on this scale. A new measurement technique is being investigated at the BNL-ATF by Andonian *et al*.^44^. Such a measurement would use a TEM_01_ mode laser and a resonant undulator to impart a transverse kick on the scale of the laser wavelength and then employ an RF deflector for a transverse kick on the scale of the RF. Producing a measurement of the IFEL microbunching with this so-termed “attoscope” technique is a future goal for the UCLA-BNL team.
